# Seasonal challenges for a California renewable- energy-driven grid

**DOI:** 10.1016/j.isci.2021.103577

**Published:** 2021-12-07

**Authors:** Mahmoud Y. Abido, Zabir Mahmud, Pedro Andrés Sánchez-Pérez, Sarah R. Kurtz

**Affiliations:** 1 Mechanical Engineering Graduate Program, School of Engineering, University of California Merced, Merced, CA 95343, USA; 2 Aerospace Engineering Department, Faculty of Engineering, Cairo University, Giza, Cairo 12613, Egypt; 3 Environmental Systems Graduate Program, School of Engineering, University of California Merced, Merced, CA 95343, USA; 4 Materials and Biomaterials Science and Engineering Graduate Program, School of Engineering, University of California Merced, Merced, CA 95343, USA

**Keywords:** Energy resources, Energy policy, Energy sustainability

## Abstract

Currently, the most difficult time of year for California to supply the demanded electricity is around sunset on very hot summer days. As California uses more renewable electricity, that challenge may shift to any time of the year depending on the supply of electricity more than on the demand. We study various scenarios for applying a 100% renewable energy grid using six years (2015–2020) of historical demand and scaled-up solar and wind generation to investigate the main function of the storage in affording adequate electricity supply at all times of the year. We identify the times of year that may be most challenging. We detect that, for a solar dominant generation profile, the ultimate challenge shifts from summer to winter. Furthermore, the critical time of the year may be shifted by one or two months depending on the amount and the mix of the renewable generation that will be built.

## Highlights

• Winter will be the challenging season for a solar-dominated grid in California
• The challenging date shifted from March to January by increasing the generation
• Building 30% more generating capacity may halve the total storage needed

## Graphical abstract

**Figure:**
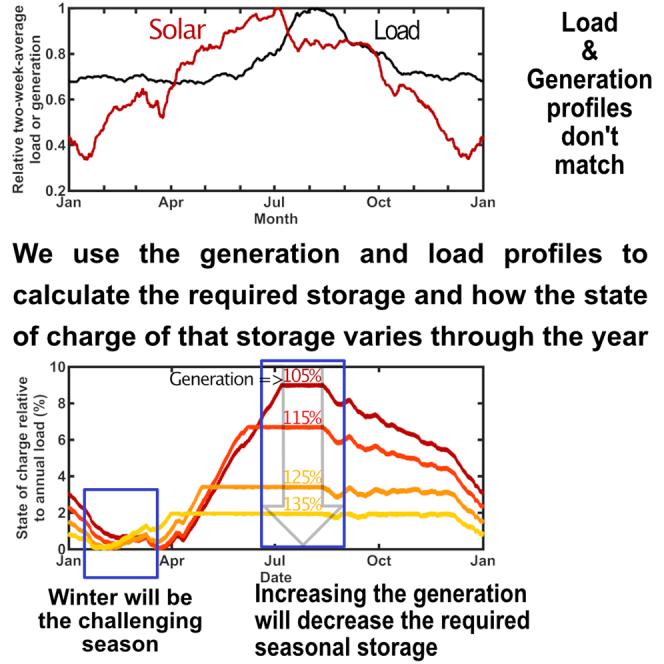

## Introduction

Adequate supply of electricity to maintain reliable grid function will be a key element for successful implementation of a renewable-energy driven grid. Decarbonizing the electricity grid (Lombardi et al., 2020; Denholm et al., 2021; Victoria et al., 2021; Tröndle et al., 2020) is a long-term target for a growing number of countries. During the widespread heat wave in California in August 2020, resource inadequacy around the time of sunset forced California Independent System Operator (CAISO) to cut electricity supply to customers (California Independent system Operator (CAISO) et al., 2021). Such events raise questions about the practical penetration level of variable electricity sources (solar and wind) and have motivated much discussion (California Independent System Operator (CAISO), 2021c) about CAISO's ability to meet the demands in the coming years, especially when the Diablo Canyon nuclear plant is scheduled to be decommissioned by 2025 and the availability of imports may be reduced during critical hours as nearby states rely more on renewable electricity. Similar challenges are anticipated around the world as the use of variable solar and wind electricity generation increases.

Resource adequacy for a fossil-fuel powered grid may be met by installing relatively inexpensive peaker plants that are anticipated to sit idle for much of the year and then operated only during times of high demand. In California, during times of acute shortages, prices may increase to $1000/MWh (Hundiwale et al., 2019), enabling the investors in the peaker plants to receive substantial income during those short times. As solar and wind electricity become key sources of electricity, battery storage is becoming increasingly important toward meeting instantaneous demand. Mallapragada et al. predicted that 4% lithium-ion storage would be needed for 40%–60% penetration of solar and wind (Mallapragada et al., 2020). In 2021, close to 30% of electricity generation in California will be from solar and wind and the state is routinely providing 2% of power from batteries during times of peak demand, consistent with Mallapragada's prediction. Resource adequacy for a renewable-energy driven grid requires resources to deliver the peak power and, to the extent that those resources use stored energy (inclusion of nuclear power and fossil generation with carbon capture and sequestration are possible approaches and largely avoid the need to consider the stored energy, but are outside of our scope, which focuses on a renewables-driven grid), adequate stored energy must also be available. The dual focus on both power and energy for a renewable-energy-driven grid represents a change in the discussion of resource adequacy (Parks, 2019). Thus, the methods typically used to meet resource adequacy in a fossil-fuel powered grid differ substantially from those relevant to a grid supplied by renewable resources, focusing more on how variable weather affects generation instead of how variable weather affects demand (Dowling et al., 2020; Shaner et al., 2018).

Studies have identified times when a lack of solar and wind over several days or weeks will limit the ability of high levels of solar and wind to provide resource adequacy. Shaner et al. (Shaner et al., 2018) found that several weeks of energy storage would be needed to get through variable weather in a solar and wind-driven grid unless solar and wind plants are built to supply surplus electricity. Dowling et al. (Dowling et al., 2020) showed how long-duration storage (with lower costs associated with increased energy capacity) could help to address times when solar and wind electricity would be unavailable. Rinaldi et al. extended that study to focus specifically on California, finding that when California was treated as an island, costs could be reduced by 21% by using long-duration storage (Rinaldi et al., 2021). Tarroja et al. also considered California with a 100% renewable energy electricity system (Tarroja et al., 2018). Although Tarroja's focus was on the materials usage, their calculations shed light on the question of the most difficult times to retain adequate energy in storage, concluding that storage will fill during the summer and reach low levels during the winter for the scenarios they presented.

Here we build on our previous study (Abido et al., 2021), which demonstrated that building many solar plants could easily supply the needed electricity during the summer, but that stored energy might run low during the winter without an adequate storage reservoir. We use an energy balance approach to identify the seasonal storage challenges that California (and other similar locations) may anticipate if a renewables-plus-storage approach is used to reach a zero-carbon-emissions grid. We start by reviewing the resource mix that California may be able to access and why it may experience a seasonal challenge that is not found in many locations. Then, we present results showing how energy balance— that is, energy in and out of storage is affected by the selected scenario, and describe how the time to be most concerned about resource adequacy in California will change from what it is today for plausible renewable-energy-driven scenarios. In the STAR methods section, we describe our energy-balance approach which assumes that practical (i.e., low-cost and efficient) storage is available and perfectly connected. In the method details section, we present a flow chart for the in-house python code that is used in this study.

## Results and discussion

### Background – resource and technology availability

**Figure 1:**
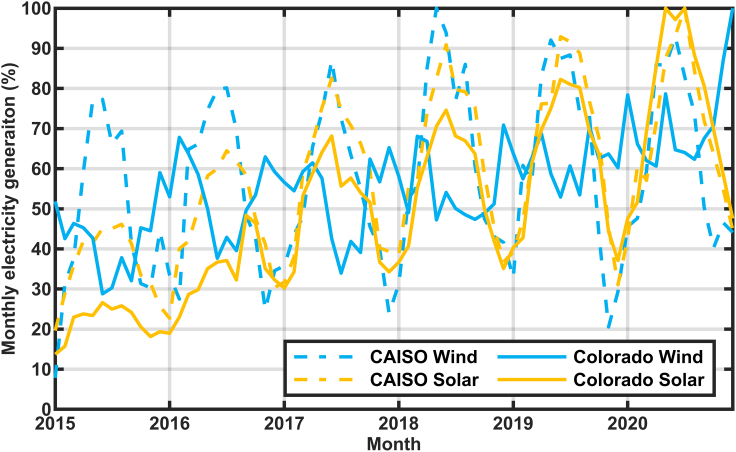
Monthly electricity generation from solar and wind in California (CAISO) and Colorado.

Although storage may be used on short time scales, here we focus on seasonal storage to answer the question “what times during the year will we be most concerned about resource adequacy?” We seek to answer this question in the context of a renewable-electricity-driven grid in sunny locations like California. The need for seasonal storage in a renewable-driven grid may be avoided in many locations by adjusting the relative installation of solar and wind power plants (Becker et al., 2014; Heide et al., 2010; Budischak et al., 2012; Weschenfelder et al., 2020; Slusarewicz and Cohan, 2018). Figure 1 compares historical monthly solar and wind electricity generation in California (as reported by California Independent System Operator (CAISO), 2021b) and Colorado (as reported by U.S. Energy Information Administration (EIA), 2021a). As expected, the solar electricity generation is at a minimum around January of every year. Least expected, the historical California wind-generated electricity also shows a minimum in January or during winter months. In contrast, wind in Colorado tends to increase during the winter. In both locations, the wind tends to blow more at night, allowing it to complement the daytime solar electricity very well, but the Colorado wind is much better than the California wind in complementing the seasonality of the solar electricity generation.

Thus, a renewable-driven grid in Colorado may select an optimal ratio of solar and wind to meet the year-round demand in a more consistent way. In contrast, balancing solar with wind does not decrease seasonal storage needs when the generation profiles look like those shown in Figure 1 for California. California may benefit from importing wind from locations like Colorado. In addition, there may be locations onshore within California that could provide stronger wind resources during the winter (Mahmud et al., 2022). Alternatively, offshore wind may provide more consistent electricity generation.

Offshore wind speeds in California also decrease during the winter, but the offshore wind speeds are higher than onshore wind speeds, resulting in more consistent wind generation throughout the year (Dvorak et al., 2010). California is discussing installation of offshore wind starting in 2026 (Chiu, 2021). California's coast has very little opportunity for wind in shallow areas; therefore, floating platforms will be needed, increasing the cost and the risk, but there is substantial potential as well as substantial interest (Beiter et al., 2020). Nevertheless, the available resource for both onshore and offshore wind is estimated to be limited (California Public Utilities Commission (CPUC), 2018) suggesting that it will be difficult to find enough economically attractive sites to enable an optimal balance between solar and wind generation.

Today, geothermal and biomass plants are typically operated in California with a constant output, though it may also be possible to operate these as flexible generators (Millstein et al., 2020). These could be helpful in meeting winter load, but in 2020, the electricity generated by geothermal and biomass were 3.7% and 1.2%, respectively, out of the total generation reported by CAISO (California Independent System Operator (CAISO), 2021a, 2021b). The use of biomass is not anticipated to grow substantially because of the low availability of low-cost feedstocks and because of the high cost of collecting materials. However, there is a possibility that the need for reducing fuel in forests to reduce the severity of wildfires will motivate investment in collecting forest waste, allowing electricity generation from those materials to become cost effective. A possible estimate for that potential may assume the availability of about 50 million tons of biomass per year (Baker et al., 2020). If this biomass can generate electricity with a higher heating value of 15 MJ/kg with 25% conversion efficiency, about 50 TWh can be generated from California's biomass each year. Use of biogas from landfills and installations of digesters at waste-water treatment plants is increasing under incentives such as the Low Carbon Fuel Standard (California Air Resources Board, 2021), supporting the possibility of reaching the 50 TWh/year generation potential; however, biogas is not increasing fast enough to motivate inclusion of these levels in modeling (California Public Utilities Commission (CPUC), 2018).

Similarly, geothermal power generation is found to be relatively expensive and unlikely to expand by even a factor of two (California Public Utilities Commission (CPUC), 2018). However, investment from the oil and gas industries (Brigham, 2021) could rapidly reduce the cost. If cost reduction were achieved, the resulting geothermal resource could provide ample power (Tester et al., 2006).

Hydropower can play the dual roles of generation and storage (Office of Energy Efficiency and Renewable Energy, 2021a; Anon, 2017). It may directly (as pumped hydro) or indirectly (by controlling output) act as a storage. However, in a dry year, it may not contribute much and in a wet year it may need to be used in a continuous manner to provide stable flow in the rivers or may need to be used when the reservoirs fill, limiting its ability to match supply and demand, especially in a reliable way.

The conclusion that solar and wind are the primary available resources is common for many locations around the world (Victoria et al., 2021), especially because of the low costs that solar (Haegel et al., 2017) and wind electricity have now reached (Ellabban et al., 2014), enabling them to compete with fossil-fuel electricity. The lack of wind to complement solar resources is also found in, for example, Florida, India, and most places near the equator. Although each location will vary in its needs, the approach we present here may be applied to most locations and the conclusions will be similar, to the extent the available renewable electricity resources are similar.

To be cost effective and reliable, a 100% renewables-driven grid will require a large amount of storage (Dowling et al., 2020; Shaner et al., 2018; Tong et al., 2020). Here, we have assumed adequate availability of storage at an acceptable cost without attempting to identify the source of the technology. Storage technology is evolving rapidly with many innovations being explored (Kittner et al., 2019; Schmidt et al., 2017; Shan et al., 2022). Although the weather dependence of solar and wind are critical to defining the challenging times of year, it is less clear that the choice of storage technology will affect the time of year when storage may be depleted. Thus, we do not attempt to define a specific set of storage technologies but create a hypothetical central storage reservoir for accounting purposes.

In the rest of the paper, we explore the impact of a range of renewables-driven scenarios on the time of year when the energy resource adequacy may be most challenged. The scenarios were chosen to explore the effects of the various possibilities, even those that are unlikely. We then discuss the implications in the context of which of the scenarios are most plausible based both on the cost and scalability of the various generation technologies, reflecting the information presented in this section.

### Energy balance model results

**Figure 2:**
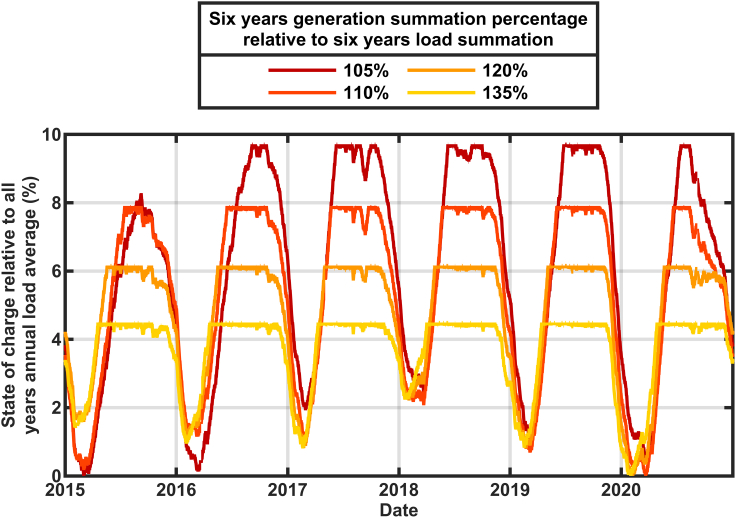
Calculated state of charge for stored energy using 2015–2020 generation and load data adjusted to reflect renewables-only grid scenarios The charging rate is constrained to 50 GW with storage round-trip efficiency of 80%.

**Figure 3:**
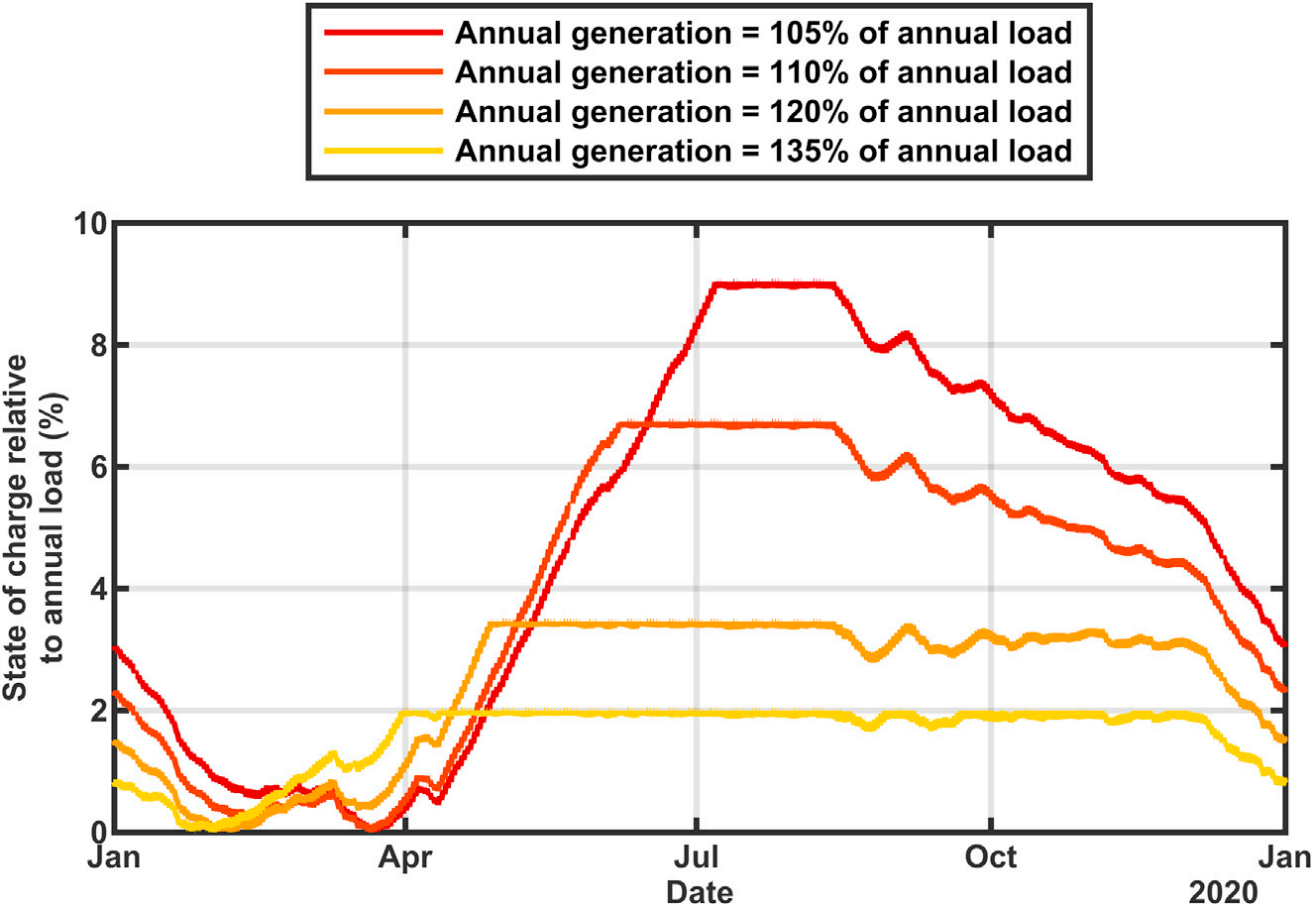
Calculated state of charge for stored energy using 2020 generation and load data adjusted to reflect renewables-only grid scenarios The charging rate is constrained to 50GW with storage round-trip efficiency of 80%.

The effect of the size of the solar buildout on the calculated state of charge is shown in Figures 2 and 3. These calculations used data for 2015–2020 in Figure 2 and only 2020 in Figure 3 to show more detail. The historical thermal generation, nuclear generation, and imports (California Independent System Operator (CAISO), 2021b) were replaced with additional solar generation according to Equations (1a) and (1b)

(Equation 1a) AddedPower=AddedbuildFactor×HistoricalResourceGeneration

(Equation 1b) TotalGeneration=AddedPower+Hydro+Renewables

using the solar multipliers in Table 1 to achieve a total annual generation to total annual load of 105%, 110%, 120%, and 135% for each year separately. The state of charge is graphed as a percentage of the average of the six years annual loads. This six-year analysis clearly shows that for each year the time of the biggest challenge is around February. However, in some years, the storage retained much more reserve even in February. The two years that showed the lowest states of charge (2015 and 2020) correlate with the smallest hydropower (5.4% in 2015 and 6.5% in 2020) so there is more dependence on the storage to supply the grid in those years. However, the details of which year is most challenged also depends on the amount of the solar overbuild. The scenarios with more solar overbuild show less dependence on the hydropower and more on the solar resource. Specifically, although Table 1 shows a systematic decrease in the solar multiplier during the years from 2015 to 2019, reflecting the increasing deployment of solar in California, the solar multiplier used for 2020 increased, reflecting the low solar output that was observed for that year. A more detailed inspection of the data (not shown) revealed that the solar generation was low during late 2019 as well as early 2020, causing the storage to deplete very rapidly from its filled state in summer of 2019, especially for the scenarios that were relatively more dependent on solar electricity (with high overbuild).

**Table 1:** Generation mixtures (X means multiples and % means percentage relative to the annual load)

Resources/Year		2015	2016	2017	2018	2019	2020
Solar multiplier	105%	12.1X	8.2X	6.1X	5.6X	5.0X	5.2X
	110%	12.8X	8.8X	6.6X	6.0X	5.4X	5.5X
	120%	14.3X	9.9X	7.5X	6.8X	6.2X	6.3X
	135%	16.5X	11.5X	8.9X	8.0X	7.3X	7.4X
Total added solar	105%	81.4%	73.7%	67.3%	68.8%	65.5%	70.9%
	110%	86.4%	78.7%	72.3%	73.8%	70.5%	75.9%
	120%	96.4%	88.7%	82.3%	83.8%	80.5%	85.9%
	135%	111.4%	103.7%	97.3%	98.8%	95.5%	100.9%
Historical renewables		18.3%	21.5%	24.0%	26.8%	27.5%	27.6%
Large hydro		5.4%	9.8%	13.7%	9.4%	12.0%	6.5%

In Figure 3 the resulting annual generation mix to exactly meet 2020's load (≅ 220 TWh) included 79.6% solar, 7.4% wind, 6.5% hydropower, and 6.5% other renewables (geothermal, biomass, biogas, and small hydropower). The reservoir is found to reach its minimum state of charge between Jan 24 for large solar build out (annual generation = 135% × annual load) and March 21 for small solar build out (annual generation = 105% × annual load). The systematic shift in the time of minimum energy in storage is a direct result of how quickly the storage can be filled during daytime hours from the solar electricity. Greater solar build out enables the storage reservoir to begin to refill in January, whereas minimal solar build out requires March's longer days.

**Figure 4:**
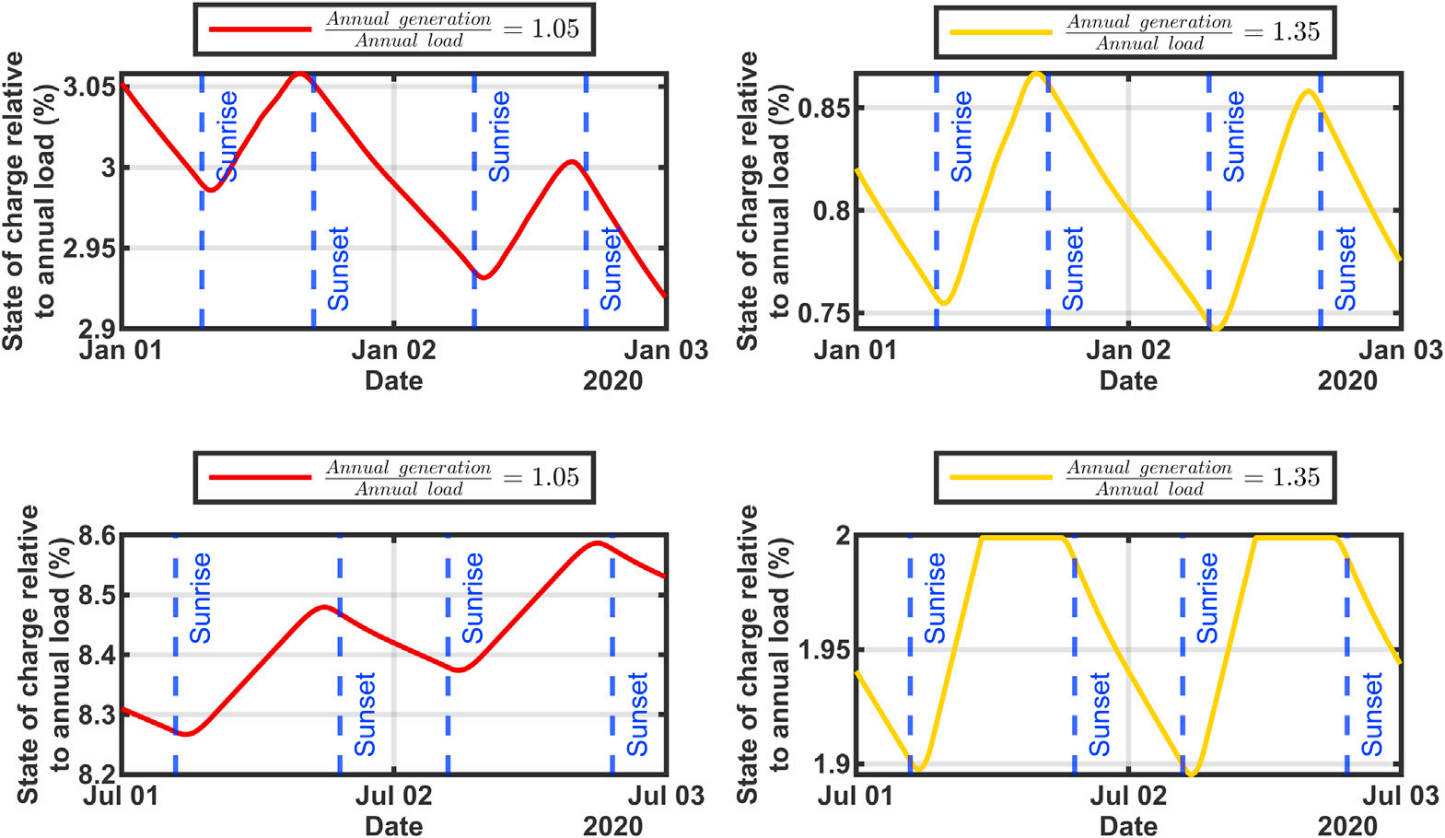
Magnifying two days of January and another two days of July to show the daily charging and discharging details

Although California’s August 2020 emergency occurred around sunset, the storage reservoir in Figure 3 reaches a minimum charge state just after sunrise, as shown in Figure 4, which expands the data from Figure 3 to view days in January and July for two levels of generation. The times for sunrise and sunset were taken for the centrally located California City. On most days, the minimum and maximum in the state of charge are observed approximately an hour after sunrise and before sunset, respectively, reflecting that the sun needs to be away from the horizon before the solar electricity generation increases enough to supply much of the load. These observations pertain to the energy balance of California's entire grid with generation dominated by solar generation. The times of day for the minima and maxima are expected to vary with the weather, location, and the technology mix used for the generation.

**Figure 5:**
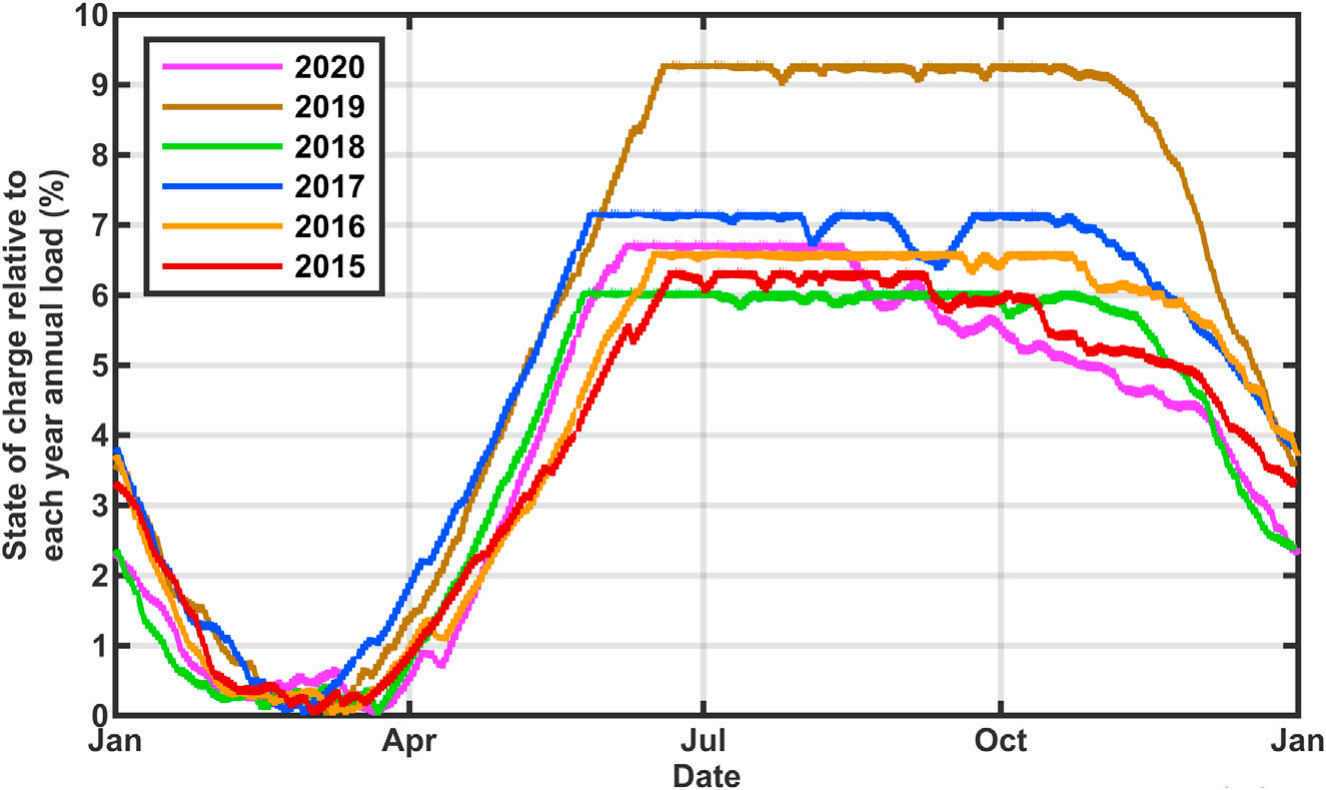
Calculated state of charge for stored energy using data from 2015 to 2020, but showing only the total annual generation = 110% of annual load case for each year

Similar calculations for 2015–2020 (Figure 5) showed that the minimum state-of-charge in the reservoir is always observed during the winter or early spring, even in 2020 which experienced lower than usual solar generation because of wildfires and cloudy weather. Although the exact date of the minimum state-of-charge varies each year, for a given level of build out, the date of the minimum varies by less than one month, suggesting that once the build out is defined for a solar-dominated grid, the time of highest risk for resource inadequacy can be well predicted.

**Figure 6:**
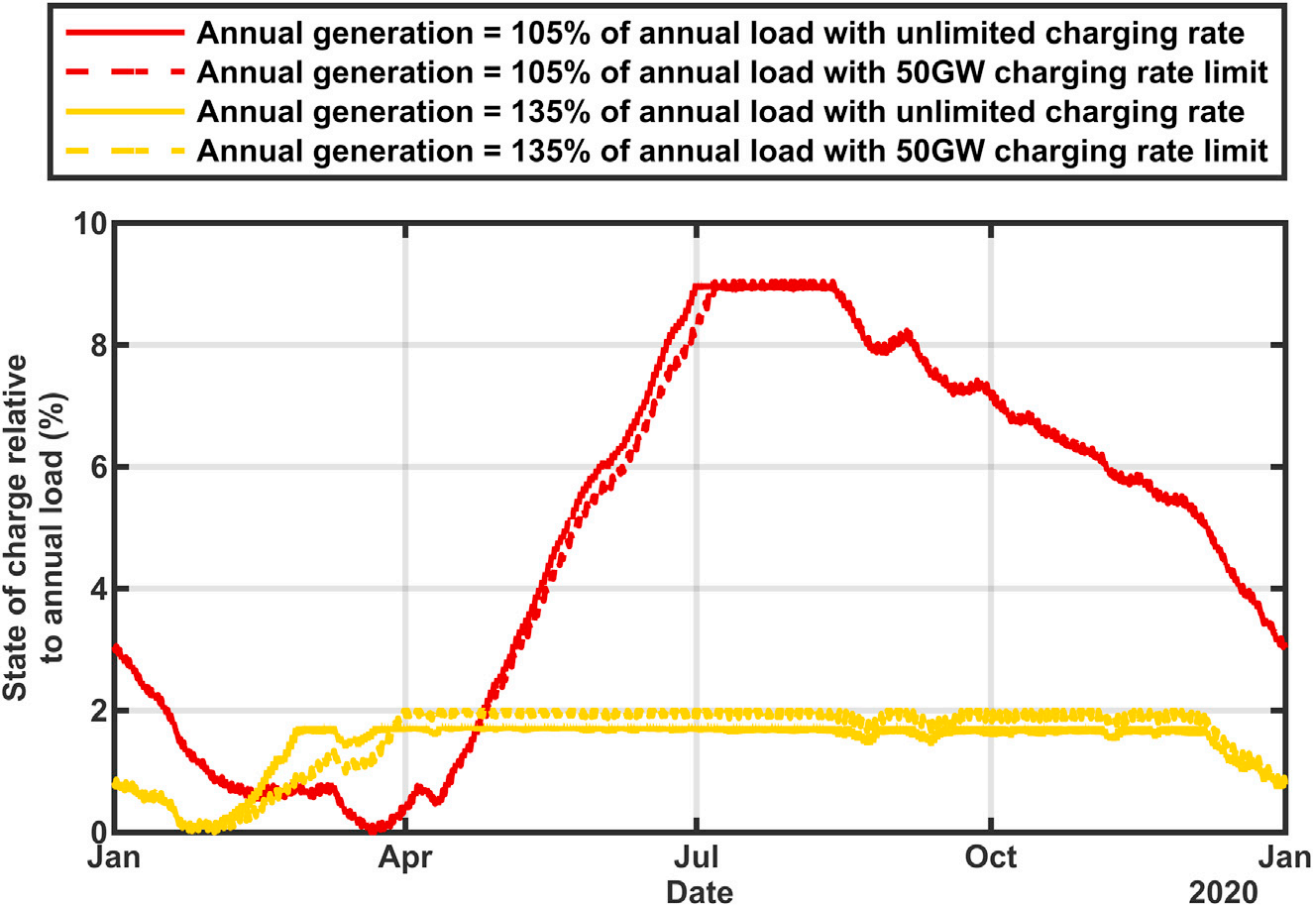
Calculated state of charge for stored energy using data from 2020, but comparing calculations when the charge rate was limited to 50 GW and unlimited, using two build out levels, as indicated

When defining a storage asset, we may consider the energy rating and the power ratings for both charging and discharging. In Figures 2, 3, 4, and 5 we have described the energy in the reservoir assuming that the charge rate was limited to 50 GW based on the maximum load during the 2015–2020 period (California Independent System Operator (CAISO), 2021d). Some types of storage reservoirs use different converters for the charging and discharging, enabling differing power ratings. In Figure 6 we show the effect of enabling higher charge rates and see that unconstrained charging has very little effect when the build out is small (105% curves) but hastens the recharging of the reservoir and decreases the storage needed slightly when the annual generation is 135% of the annual load.

**Figure 7:**
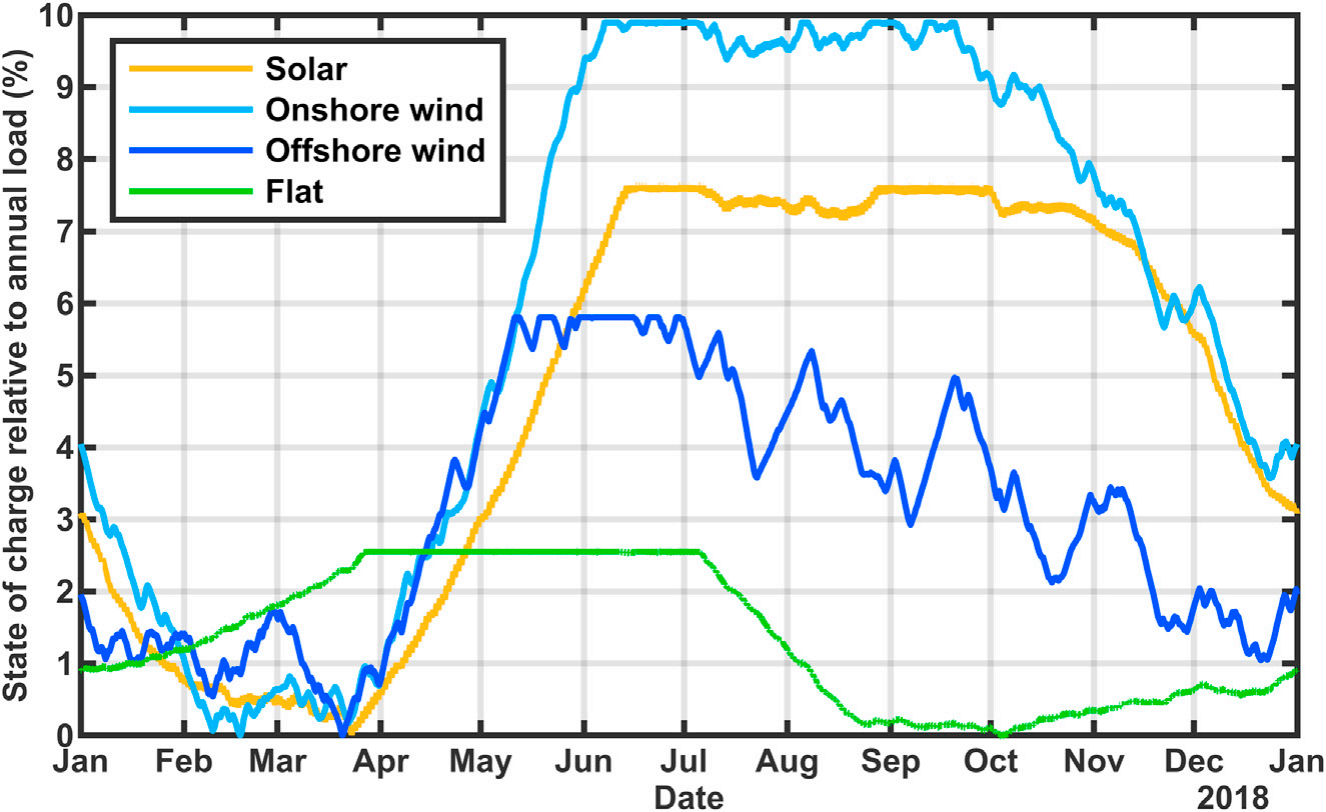
Calculated state of charge for stored energy using 2018 generation and load data with thermal, nuclear, and imports replaced with electricity generation from a single technology (as indicated) to deliver total generation equal to 105% of the annual load

It is fairly unlikely that an all-renewable grid in California will be constructed by building only solar (Shaner et al., 2018). We repeated the calculation of Figure 3, expanding the generation using the generation profiles for onshore wind (from the historical data), offshore wind (simulated), and a constant (“flat”) value. The results are shown in Figure 7. For each of these cases, the reported 2018 generation from solar, hydropower, wind, and other renewables were retained while scaling up one of the generation profiles to replace the thermal and nuclear generation with that resource. As shown in Figure 1, the California onshore wind tends to be greater in the summer compared with winter, so an even larger storage reservoir is needed. Offshore wind and flat renewables come closer to matching the load seasonally, so a much smaller storage reservoir is needed. Although adding onshore wind, solar, or offshore generation results in the minimum storage level in February or March, a similar build out with a flat generation profile results in the minimum shifting to October and extending for a couple of months after the high load in July and August depleted the storage.

**Figure 8:**
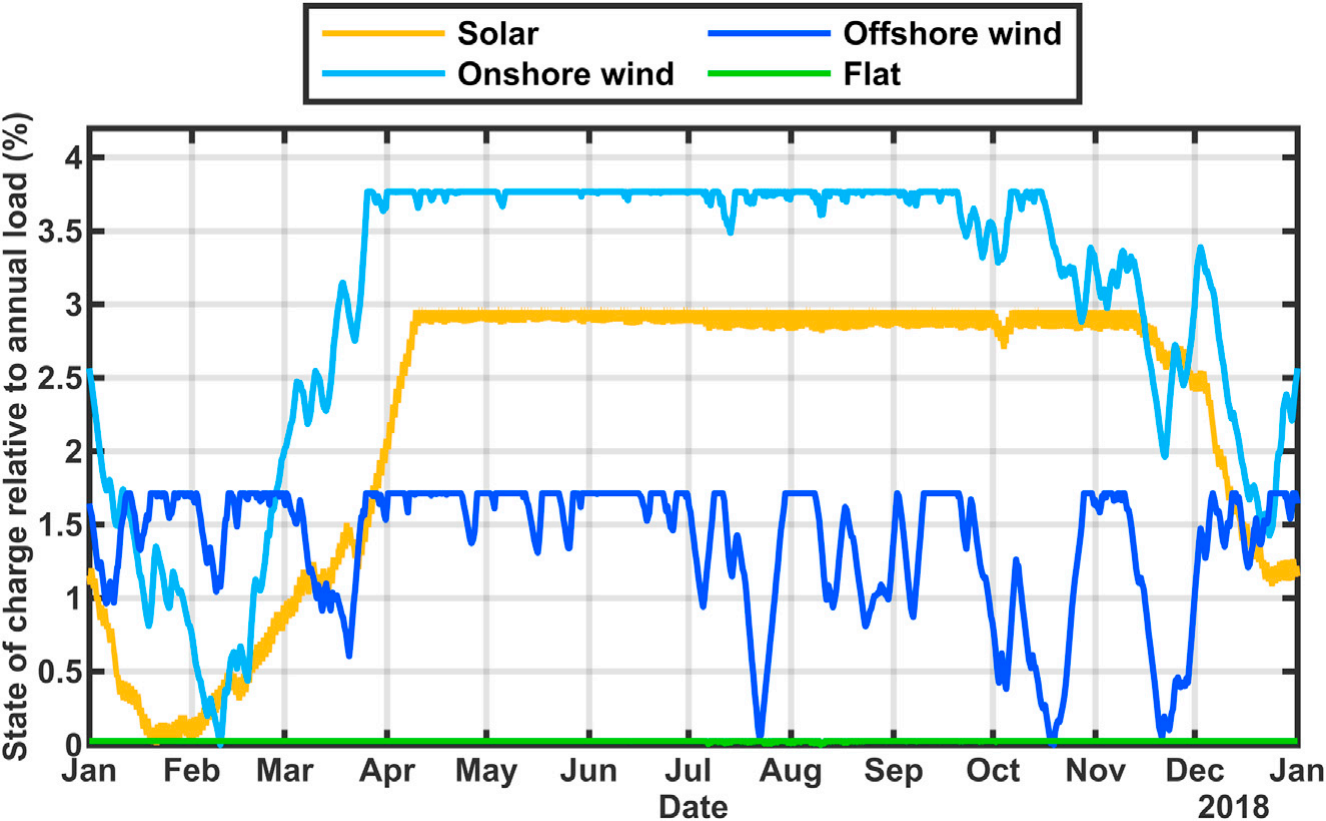
Calculated state of charge for stored energy using 2018 generation and load data with thermal, nuclear, and imports replaced with electricity generation from a single technology (as indicated) to deliver total generation equal to 135% of the annual load

If the resources are built out in a bigger way as shown in Figure 8, only the solar build out and the added onshore wind result in a minimum state of charge in winter. Again, build out of onshore wind results in the need for the largest energy reservoir. Adding a flat generation profile to meet a total annual generation equal to 135% of the total annual load resulted in adequate electricity generation at all times. In the case of the offshore wind build out, the reservoir reaches near zero at times ranging from July to November, or throughout the year for other years, reflecting the greater variability of the offshore wind resource.

**Figure 9:**
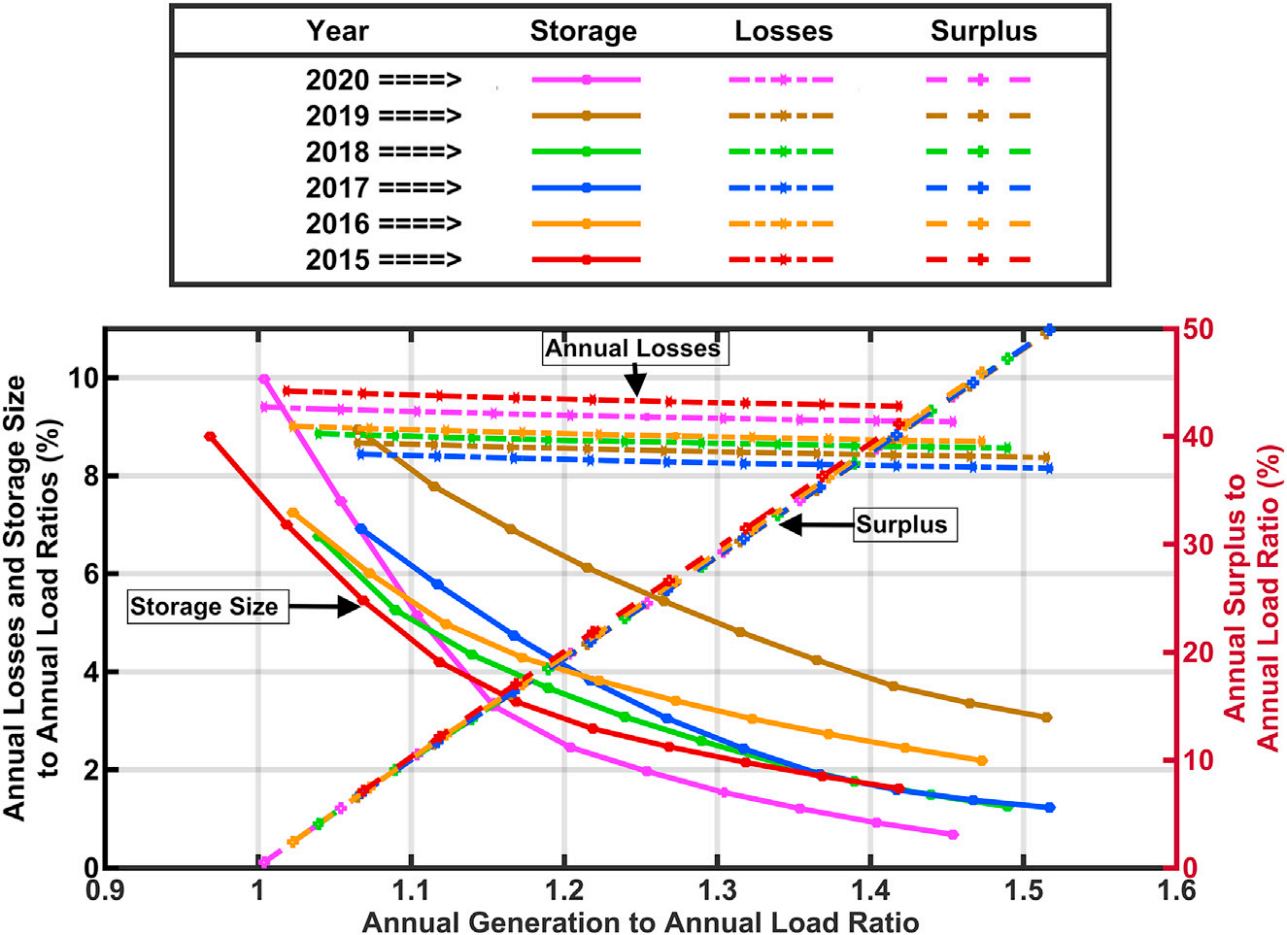
Storage needed to meet minimal resource adequacy and the losses due to storage round-trip efficiency (left axis) and associated surplus electricity (right axis) as a function of solar build out

In addition to a shift in time for when the minimum state of charge is observed, Figures 2 and 3 show that the needed storage reservoir decreases as the solar generation is increased, as would be expected, and as shown in Figure 9 for years 2015–2020. Figure 9 also shows how the surplus electricity increases linearly (by design) with the annual generation as the solar generation is increased. We suggest that this “surplus” may be used for the transportation sector, the chemical sector and other energy demands. If, for example, the “surplus” electricity was used to make hydrogen for production of fertilizer and to fuel trucks, steel making, and furnaces, the demand for the “surplus” electricity might be substantially greater than what we have described. In that case, resource adequacy concerns could be met by providing low electricity prices to the companies using the “surplus” electricity in return for their promise to stop using the electricity whenever the generation is challenged to meet the current load. An analysis of the feasibility of directing this surplus electricity (which is mostly generated during the times of the year when the storage becomes full as shown by the ‘plateaus’ in Figure 2) is beyond the scope of this paper, but we note that the United States Energy Department's “Solar Futures” study suggests that the United States will need 1.6 TW of solar for a decarbonized grid and an additional 3 TW of solar to decarbonize the other energy sectors (Office of Energy Efficiency and Renewable Energy, 2021b). Thus, the need for energy for other energy sectors (which may be more flexible in its timing) is likely to be larger than the energy needed for the power sector and these cross-sector applications will benefit from even more expansion of the generating capacity than we have modeled here. An even greater build out of solar energy plants would further reduce the size of the needed storage, but would have a smaller effect on the time of year when the storage would reach its minimal state of charge.

The <10% losses we show in Figure 9 are relatively small. If daytime loads can always be met directly, while nighttime loads require storage, then roughly half of the delivered electricity will suffer the inefficiency of the charging and discharging. Thus, we may expect that the losses should always be a little less than half of the round-trip charging loss, as reported here.

**Figure 10:**
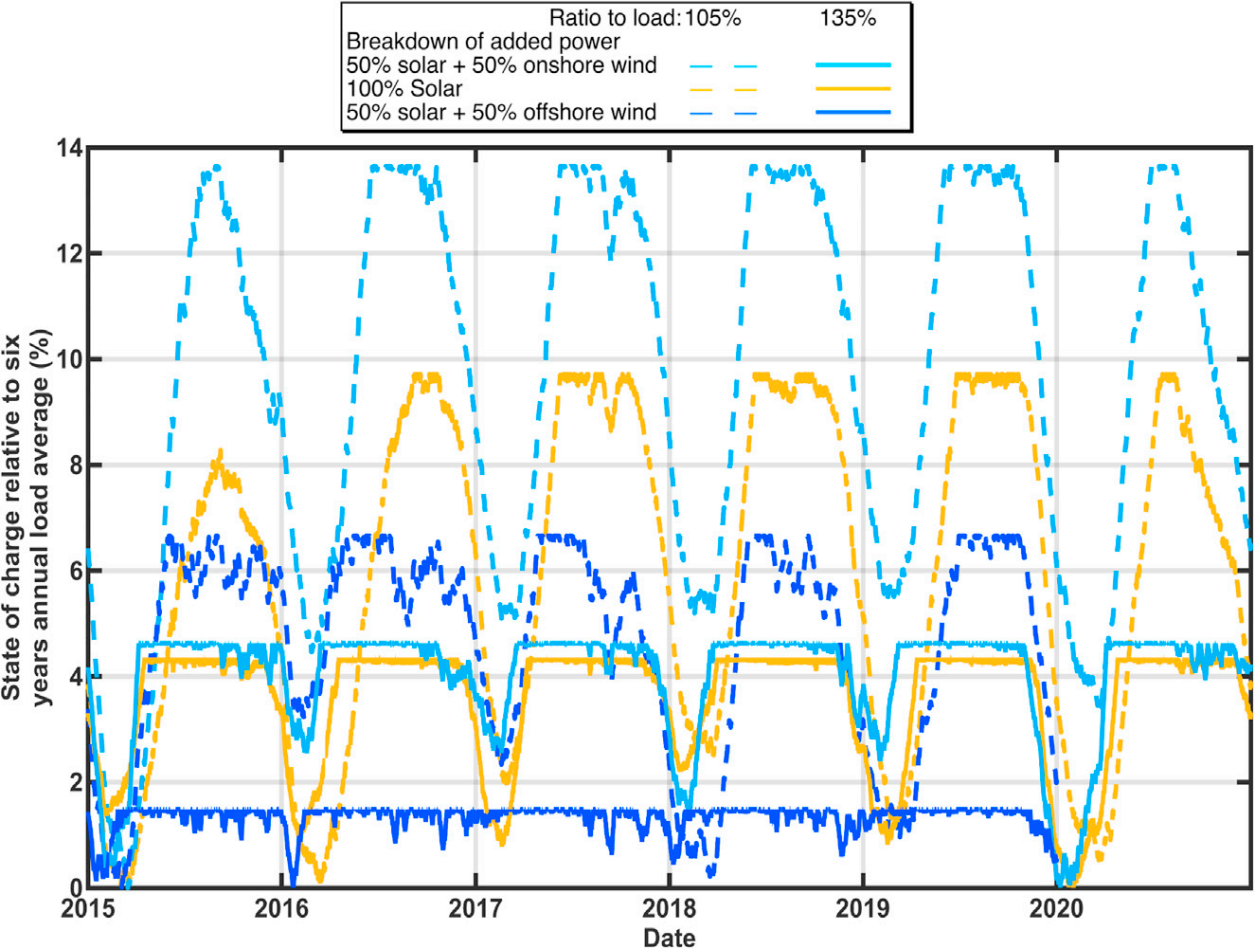
Calculated state of charge for stored energy using renewables generation and load data for years 2015–2020 adding additional solar and wind generation as indicated in the legend to replace thermal, nuclear, and imports Offshore wind speed data were not available for 2020.

A future renewable-energy driven grid in California is likely to include a mixture of technologies, rather than expanding a single technology, as shown in Figures 7 and 8. The effect of adding wind alongside solar is shown in Figure 10, comparing the addition of 1) equal amounts of solar and onshore wind, 2) only solar (as for Figures 2, 3 and 5), and 3) equal amounts of solar and offshore wind. In general, adding the mixture of onshore wind and solar required a larger reservoir, whereas a mixture of offshore wind and solar required a smaller reservoir compared with all-solar additions. The calculations are done similarly to Figure 2 but for the resource combinations shown in the legend. The results of Figures 2 and 10 show some similarities and some differences. Notably, the scenario that uses offshore wind at a high level shows highly variable times of year for each year's minimum state of charge. This is more consistent with the common assumption that renewable-energy grids need to analyze resource adequacy for all times of the year (Parks, 2019). Whether the 50% offshore wind scenario is plausible is debatable. We estimate that this scenario would require about 13 GW of offshore wind, more than is planned. Consistent with Fig 2, Fig. 10 appears to show that the years of 2015 and 2020 would have had the lowest charge states, probably caused in part by those years being low in hydropower.

Based on our analysis, we anticipate that, as more renewable electricity generators are installed, California will use more solar than wind and little more geothermal or biomass (which are currently about 4% of total generation). We anticipate that load profiles will change as electrification is increased. Electrification of heating applications will increase demand during the winter, just when a solar-driven system is already under the most stress. Electrification of the transportation sector will have much less effect on the seasonal challenges. If capabilities are developed for geothermal, biomass, and/or for hydropower to be able to be dispatchable, the need for storage will be greatly reduced. Alternatively, California may choose to add nuclear, natural gas coupled with carbon sequestration, hydrogen-powered generation, or a number of other technologies to the renewable-driven scenarios studied here. These fully dispatchable technologies may play the role of the storage reservoir studied here, or may be used more like how dispatchable thermal plants are being used today. Each of these will contribute to provide the needed resource adequacy, probably in ways that are more similar to how resource adequacy is handled. We have omitted these from our study because of our desire to understand what would be needed by a renewables-driven grid.

### Conclusion

Exploring the question “When during the year will resource adequacy be most challenged for a renewable-electricity-driven grid in sunny locations like California?” We find the highest risk times to be around sunrise during January, February, or March, depending on the amount of solar generation that is built. The renewable-energy-driven scenarios we explored show that the technology mix can have a large effect on the times of year when there is risk of resource inadequacy. However, based on the premise that California will use more solar than wind and that the current wind generates more electricity in summer than in winter, we conclude that the most challenging time will always be in the winter.

As more solar electricity is made available, the time of the seasonal challenge shifts from March to January. None of the plausible scenarios calculated the storage to reach <10% of full charge during spring or summer. On the other hand, addition of substantial wind generation at a fairly unlikely level may result in risk of the reservoir running too low at almost any time of year.

The seasonal storage needed to balance supply and demand may be cut in half by building 30% more electricity generating capacity as shown by our comparison of building generation to provide a total annual generation that is 135% vs 105% of the total annual load over a year. The surplus from the added electricity generation is anticipated to be not only useful for reducing the needed storage, but may turn out to be essential for generating hydrogen for transportation, heating, chemical, or other applications.

The effects of electrification on load profiles were not included in this study, but the addition of heat pumps to the load profiles is likely to further exacerbate the resource adequacy challenge during winter, suggesting even stronger confidence in our assertion that resource inadequacy challenges of a renewable-driven grid in California will occur in winter around sunrise. We expect similar conclusions for other low-wind, sunny locations like Florida and India, though the details will vary. The conclusions would be changed for locations with stronger wind generation during the winter and for zero-carbon grids that are not primarily driven by solar electricity. The energy-balance approach provides a straightforward method based on realistic data for exploring a wide range of scenarios.

### Limitations and assumptions of the study

This study can be expanded to include multiple features in a future study. Currently, this approach gives realistic results in that the generation and load profiles are based on observed data from the years 2015–2020 in the CAISO zone. We calculate the state of charge of the storage reservoir as a function of time of year to demonstrate the effects/trends of the following:

1. The amount of solar electricity generation
2. Limiting the charging rate
3. Other renewable electricity generation resources usage

while including a realistic round-trip efficiency for the storage. We focus on how these choices affect the time of the year when resource adequacy may be most challenged. We also explore how the size of the needed storage reservoir, the amount of surplus electricity generated and the losses due to storage round trip efficiency are interrelated.

However, this approach does not include the following:

1. Transmission constraints considerations (we balance the supply and demand for the state of California not locally) including power flow limitations, operational limitations like ramp-up-rate limitations, etc.
2. Adjusting the hydro generation to better meet the supply/demand imbalances
3. Adjusting the load profile, which may be driven by electric vehicle (EV) adoption, demand management, heat pump adoption, and many other things
4. Modeling generation profiles of the future that may differ from the historical profiles, especially because of geographical choice and system design.
5. Detailed cost tradeoff between technology choices, including duration, efficiency, capacity, renewables overbuild, and material resources required.
6. Inclusion of non-renewable energy solutions.

## STAR★Methods

### Key resources table

**Table:** 

REAGENT or RESOURCE	SOURCE	IDENTIFIER
Deposited Data		
California Independent System Operator (CAISO) generation and demand data	CAISO	http://www.caiso.com/informed/Pages/ManagingOversupply.aspx
California Independent System Operator (CAISO) monthly renewables performance report	CAISO	http://www.caiso.com/Documents/MonthlyRenewablesPerformanceReport-Jan2021.html
California Independent System Operator (CAISO) peak load history	CAISO	https://www.caiso.com/documents/californiaisopeakloadhistory.pdf
Offshore Wind Speeds	National Renewable Energy Laboratory (NREL) WindTool Prospector	https://maps.nrel.gov/wind-prospector
Monthly Net Generation United States for all sectors	U.S. Energy Information Administration (EIA)	https://www.eia.gov/electricity/data/browser/

### Resource availability

#### Lead contact

Further information and requests for resources and reagents should be directed to and will be fulfilled by the lead contact, Mahmoud Abido (mabido@ucmerced.edu).

#### Materials availability

This study did not generate new unique reagents.

### Method details

Our energy-balance approach provides a straightforward way of quantifying seasonal challenges to supplying energy when it is needed. In the end, many factors should be considered in determining the optimal technology mix (Beuse et al., 2020), but being able to generate (and store, if needed) enough electricity to meet the load in real time is foundational to every solution. All selected scenarios use historical renewable electricity generated in California to meet California’s electrical load. It is useful to use historical data as these can differ from simulated data as can be seen if one compares simulated wind data for California (Rinaldi et al., 2021) with the observed wind data for California. Importing and exporting of electricity is neglected as we focus on the worst-case situation of needing to meet all demand with local resources and follow the currently observed trend that California is increasingly less able to import electricity during times of high load (Rothleder et al., 2021).

**Figure 11:**
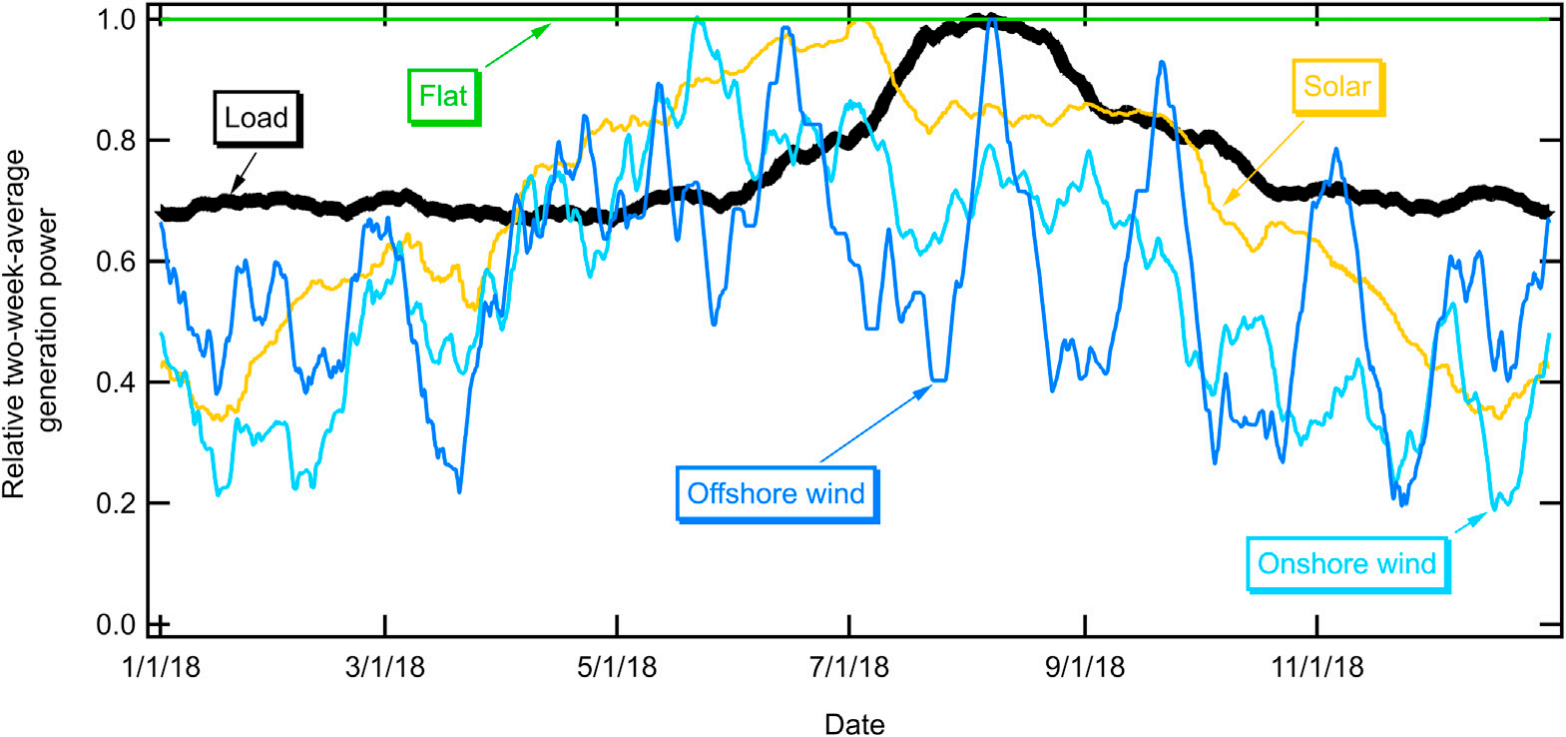
Relative generation and load profiles taken from CAISO database for 2018 with simulated offshore wind data

The generation profiles for solar, wind, and hydropower electricity were taken using historical CAISO data (California Independent System Operator (CAISO), 2021b) for years 2015 - 2020. To ensure that air conditioning and other weather-dependent loads realistically align with the solar and wind generation profiles, we used California load profiles from the same data sets. Figure 11 shows solar, wind, and load profiles for 2018 (a year that is representative of the typical trends). These 5-min data sets were first screened for missing and anomalous data. About 0.16% of the data were found to be missing. Some of them were short intervals (5 – 20 minutes) and others were long intervals (up to 7 hours). The short intervals were treated by linear interpolation using the previous and the next data points, while the long intervals were treated by linear interpolation using the previous and the next day’s data points in the same time intervals. The reported electricity from thermal, nuclear, and imported resources were replaced with scaled-up solar or wind using Equations (1a) and (1b), where *Added Power* is the historical generation multiplied by an added build factor (see Table 1 for a sample) and the other terms in Equations (1a) and (1b) are taken directly from the historical data (California Independent System Operator (CAISO), 2021b), as shown for 2018 in Figure 11. A flat generation profile was also included to simulate the consistent output that might be obtained from a geothermal plant or other constant output generator. For some of the calculations, offshore wind data were simulated using wind-speed data (National Renewables Energy Laboratory (NREL), 2021) at a height of 120 m for a location with a latitude of 35.03 and a longitude of -121.52. This offshore location provides higher capacity factors than the historical onshore wind generation profiles reported for California, but does not reflect the variability of the offshore wind generation profiles along California’s coast, which is outside of the scope of this paper. We modeled the wind power profile using the power curve for aerodyn SCD 8.0/168 (aerodyn engineering gmbh, 2021). Data for 2018 (California Independent System Operator (CAISO), 2021b), with each curve normalized to its maximum two-week average, are shown in Figure 11; data from 2015 - 2020 were used for the calculations shown in the rest of the paper.

When generation exceeded the load, the excess was placed in a single storage reservoir until the reservoir was full, with the overflow counted as “surplus” electricity as shown in Equation (2a). This surplus energy can be used in hydrogen production through electrolysis or can be supplied to industrial processes at a low price to provide low-cost products. When the generation was less than the load, energy was taken from storage to meet the remaining demand as shown in Equation (2b). The size of the reservoir was adjusted so that the state of charge of the reservoir at the end of the modeled time period matched that at the beginning of the time period (the time period was either one year or multiple years).

(Equation 2a) Generation=Load+Storagecharging+Surplus

(Equation 2b) Generation+Storagedischarge=Load

To be realistic, storage round-trip efficiency was assumed to be 80% with equal charging and discharging efficiencies (U.S. Energy Information Administration, 2021b). We explored the effect of losses and found that inefficiencies caused the need for more generation to keep a fixed surplus percentage, but did not significantly affect the time when the resource adequacy was challenged. The losses due to inefficiency were compensated by more generation. The generation is divided into two main parts: 1) the historical generation from the renewable resources like solar, wind and hydro, and 2) an added generation that is a multiple of one or more of the historical renewable resource's generation, depending on the generation combination we select to study. The difference between the total generation and the load at each time point gives the amount of charge that should be added to or withdrawn from the storage. The minimum state of charge was set to zero as a reference point. Unless otherwise noted, the charging rate was limited to 50 GW (the maximum discharging rate according to CAISO peak load) (California Independent System Operator (CAISO), 2021d) and the extra power beyond this limit was added to the electricity counted as surplus. The calculations were done using an in-house Python code that follows the following flow chart

**Figure:**
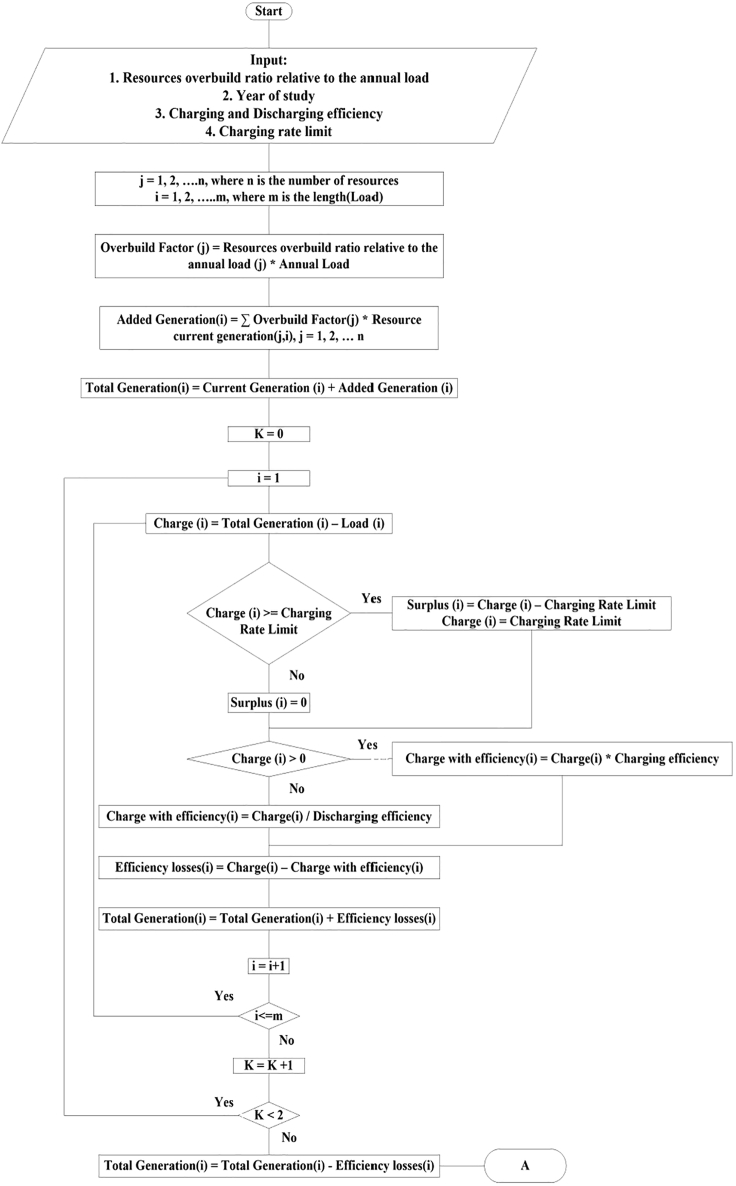

**Figure:**
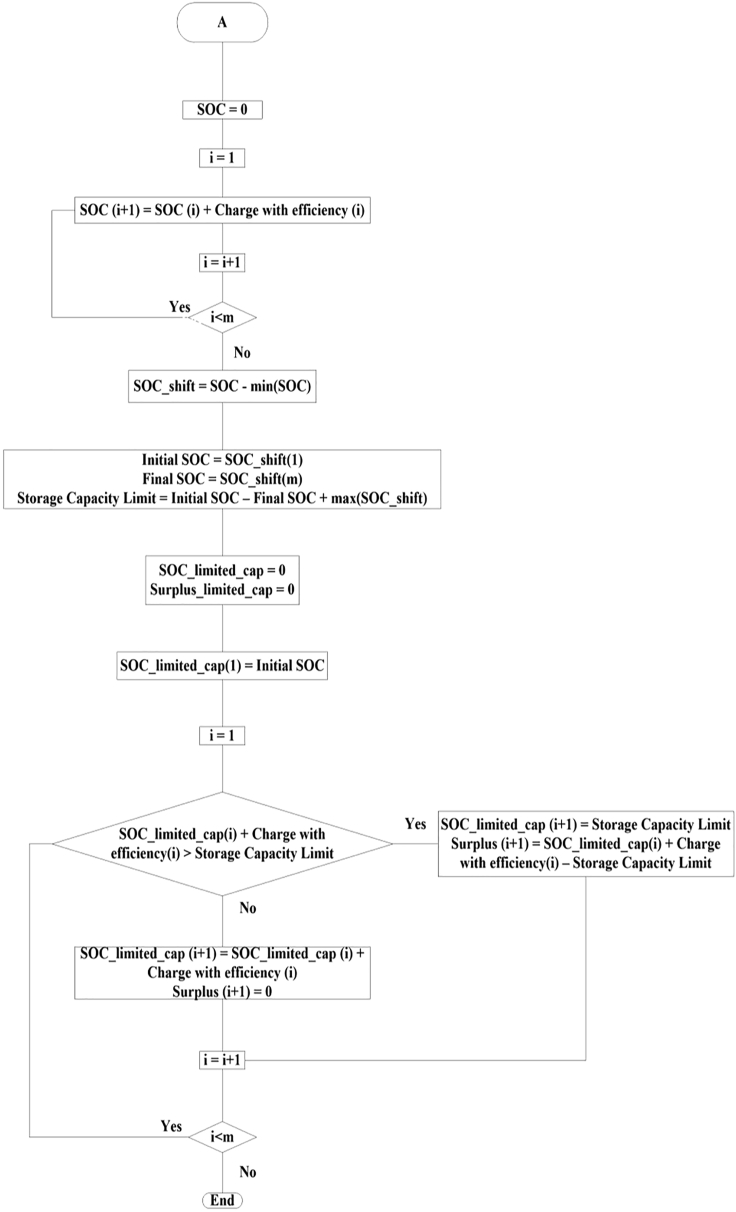

## Data Availability

• This paper analyzes existing, publicly available data which are listed in the key resources table. • Code for the energy balance approach was written in Python and is available from the lead contact upon request • Any additional information required to reanalyze the data reported in this paper is available from the lead contact upon request
